# Upregulation of microRNA135a-3p and death receptor 5 plays a critical role in Tanshinone I sensitized prostate cancer cells to TRAIL induced apoptosis

**DOI:** 10.18632/oncotarget.2152

**Published:** 2014-06-30

**Authors:** Eun Ah Shin, Eun Jung Sohn, Gunho Won, Jeong-Un Choi, Myongsuk Jeong, Bonglee Kim, Min-Jeong Kim, Sung-Hoon Kim

**Affiliations:** ^1^ Cancer Preventive Material Development Research Center, College of Oriental Medicine, Kyung Hee University, Seoul, South Korea

**Keywords:** TRAIL sensitizer, tanshinone I, apoptosis, DR5, miR135a, prostate cancer

## Abstract

Though tumor necrosis factor related apoptosis inducing ligand (TRAIL) has been used as a potent anticancer agent, TRAIL resistance is a hot-issue in cancer therapy. We investigated the antitumor mechanism of Tanshinone I to sensitize prostate cancer cells to TRAIL. Comibination of Tanshinone I and TRAIL exerted synergistic cytotoxicity, increased cleaved PARP, sub G1 population, the number of TUNELpositive cells, activated caspase 8, 9 and ROS production in PC-3 and DU145 cells. Of note, combination of Tanshinone I and TRAIL enhanced the protein expression of death receptor 5 (DR5) and attenuated anti-apoptotic proteins. RT-PCR and RT-qPCR analyses confirmed that co-treatment of Tanshinone I and TRAIL up-regulated DR5 and microRNA 135a-3p at mRNA level or activity of DR5 promoter and attenuated phosphorylation of extracellular signal regulated kinases in PC-3. Conversely, the silencing of DR5 blocked the increased cytotoxicity, sub G1 population and PARP cleavages induced by co-treatment of Tanshinone I and TRAIL. Interestingly, miR135a-3p mimic enhanced DR5 at mRNA, increased PARP cleavage, Bax and the number of TUNEL positive cells in Tanshinone I and TRAIL cotreated PC-3. Overall, our findings suggest that Tanshinone I enhances TRAIL mediated apoptosis via upregulation of miR135a-3p mediated DR5 in prostate cancer cells as a potent TRAIL sensitizer.

## INTRODUCTION

Prostate cancer is one of the most common cancers in men worldwide [1]. Most prostate cancer related deaths result from cancer metastasis, the spread of cancer from the prostate to other organs, bones and lymph nodes [2]. Though anti-cancer drugs such as Eulexin, Flutamide and Nilandron have been used for treatment of prostate cancer, their side effects have still occurred. Recent studies reported that TRAIL and microRNA (miR)s were suggested as potential biomarker or therapeutic potential in prostate cancer cells [3].

TNF-related apoptosis-inducing ligand (TRAIL) (Apo2 ligand [Apo2L]) that is a member of TNF superfamily was known to induce two death receptor DR4 (TRAIL-R1) and DR5 (TRAIL-R2) [4, 5]. There are accumulating evidences that TRAIL resulted in cell death in various cancer cell types such as colon, [6] ovarian [7] or hepatocellular carcinoma cells [8] as a selective anticancer agent.

MicroRNAs, small noncoding RNAs, play critical roles in oncogene, tumor suppressor, or apoptosis via inhibition of translation or degradation of target molecules. Recent studies showed that microRNAs modulate TRAIL induced apoptosis in cancer cells such as breast cancer or ovarian cancer [9, 10].

Cryptotanshinone, Tanshinone I, and Tanshinone IIA were known as the three major bioactive compounds from Dansen, the dried roots of the medicinal plant *Salvia miltiorrhiza* [11] that has been traditionally used for treating cardiovascular diseases [12]. Recent study reported that *Salvia militiorrhiza* with TRAIL showed evident cytotoxicity against the human lung adenocarcinoma cell line A549 and ovarian adenocarcinoma cell line [13]. Though Tanshinone I was shown to exert anti-cancer effects in non-small lung cancer [14], and breast cancer cells [15], its anti-tumor mechanism was not fully understood in prostate cancer cells.

MicroRNAs are regulated in prostate cancer and are expressed between androgen-dependent and androgen-independent metastatic prostate cancer cells [16, 17]. MiR135a is downregulated in androgene-dependent versus androgene-independent prostate cancer cells [18]. Though miR-135a functions in a tumor suppressor in several cancer cells such as renal cell carcinoma [19] or glioma cell [20], it has not fully investigated in prostate cancer cells. Thus, in the present study, the underlying apoptotic mechanism by combination of Tanshinone I and TRAIL was studied mainly in highly aggressive DU145 and PC-3 prostate cancer cells in association with upregulation of death receptors and microRNA 135a-3p.

## RESULTS

### Tanshinone I and TRAIL synergistically enhanced the cytotoxic effect in prostate cancer cells

**Fig 1:**
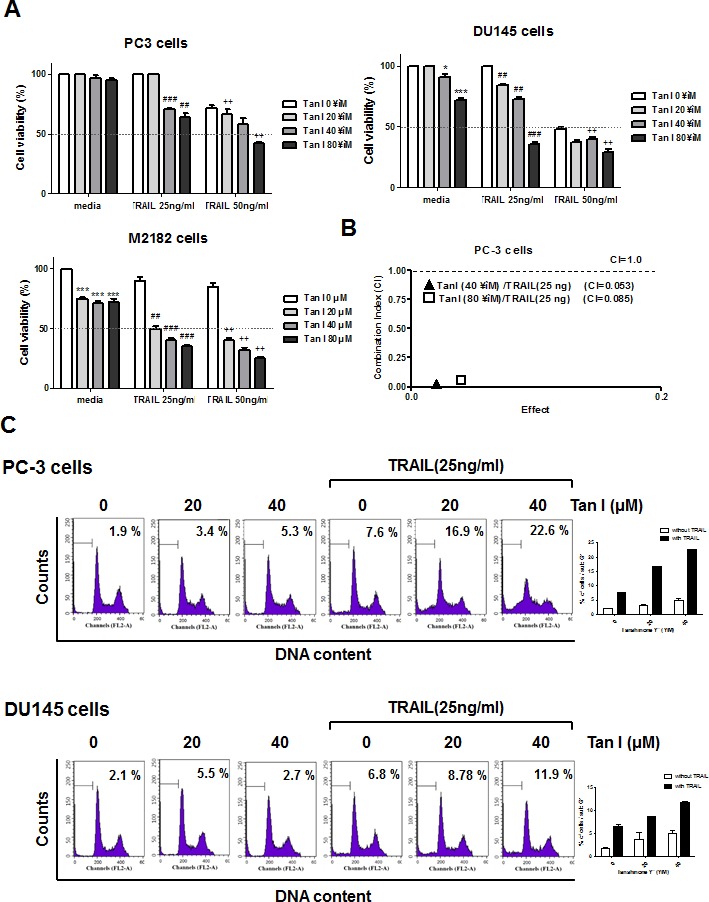
Tanshinone I enhances cytotoxicity and sub G1 population of TRAIL in prostate cancer cells (A) Effect of Tanshinone I on the cytotoxicity of TRAIL in PC-3, DU145 and M2182 cells. Three human prostate cancer cell lines were seeded onto 96-well microplates at a density of 1 × 10^4^ cells/well and treated with various concentration of Tanshinone I (Tan 1; 0, 20, 40, 80 μM) and/ or TRAIL (25 or 50 ng/ml) for 24h. Cell viability was determined by MTT assay. *p<0.05, ***p<0.001 vs untreated control, ## p<0.01, ###p<0.001 vs TRAIL 25ng treated control, ++p<0.01 vs TRAIL 50ng treated control. Data are presented as means ± SEM of triplicate samples. (B) The combination index (CI) between Tan I and TRAIL was determined by Chou-Talalay method and CalcuSyn software. (C) Effect of Tanshinone I on sub G1 accumulation of TRAIL in PC-3 and DU145 cells. Flow cytometric analysis for sub-G1 apoptotic portion in PC-3 and DU145 cells. PC-3 and DU145 cells were treated with 25 ng/ml TRAIL in the absence or presence of Tan I (20, 40 μM) for 24 h. Graphs represent percentages of subG1 portion. Data are presented as means ± SEM of triplicate samples.

To evaluate the cytotoxic effect of Tanshinone I or TRAIL, MTT assay was carried out in human prostate cancer cell lines such as PC-3, DU145 or M2182 cells. To examine the synergistic cytotoxic activity of Tanshinone I and TRAIL, various concentrations of Tanshinone I (0, 20, 40, 80 μM), and/or TRAIL (0, 25, 50 ng) were treated for 24 h in three prostate cancer cells. As shown in Fig 1A, combination of Tanshinone I and TRAIL synergistically exerted the cytotoxicity in three all prostate cancer cells. However, though M2182 cells were more susceptible to combination of Tanshinone I and TRAIL than PC-3 and DU145 cells, we performed further mechanistic study mainly in PC-3 and DU145 cells, based on previous evidences[21, 22] that PC-3 and DU145 cells were known to be more aggressive and chemoresistant to TRAIL. The significant synergy by combination of Tanshinone I and TRAIL was confirmed in PC-3 cells by using Chou and Talalay equation method, since combination of Tanshinone I and TRAIL (20 ng) showed significant combination Index (CI) values, 0.053 and 0.085 below 1 at the concentrations of 40 and 80 μM of Tanshinone I, respectively (Figure 1B).

### Combination of Tanshinone I and TRAIL dramatically induced apoptosis in prostate cancer cells

**Fig 2:**
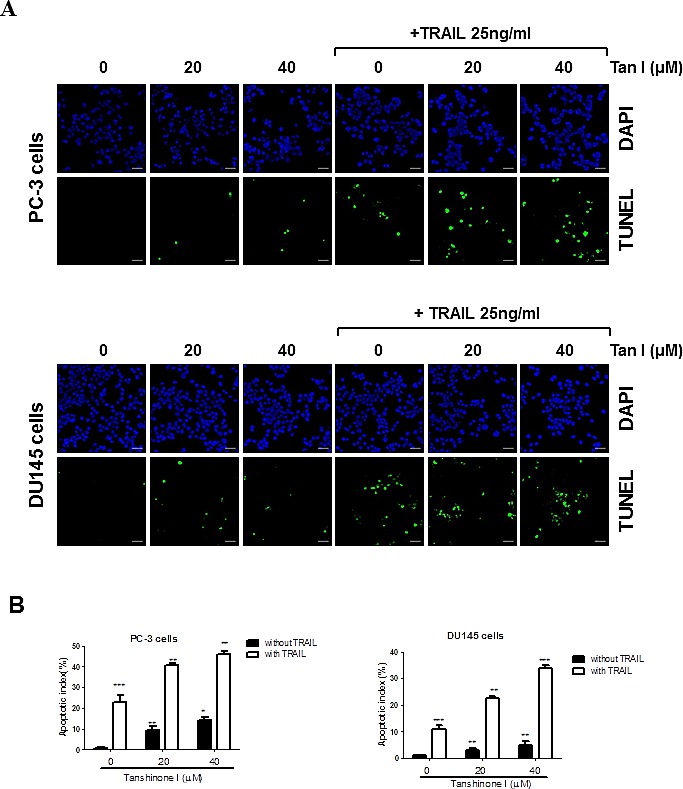

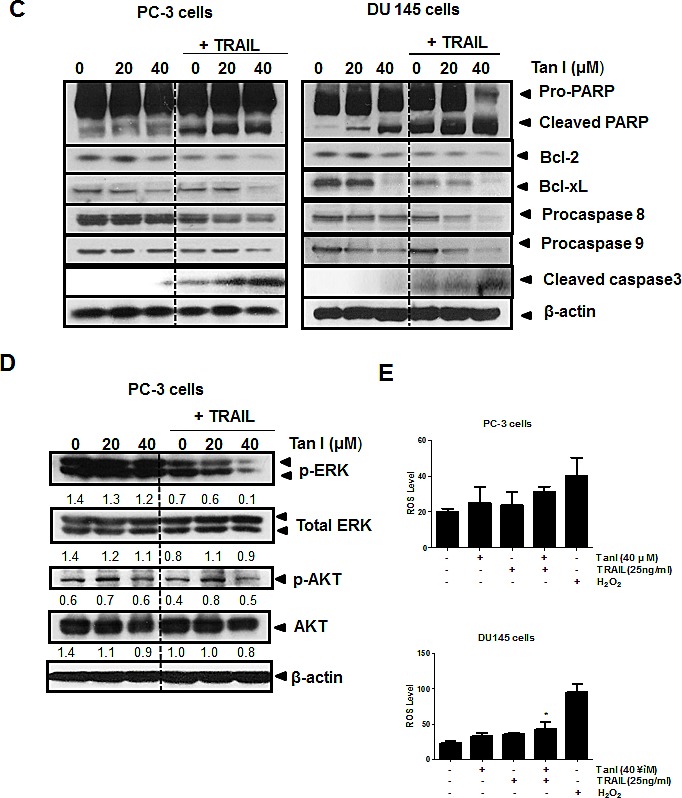
Tanshinone I regulates apoptotic proteins, generates ROS production in TRAIL treated PC-3 cells (A) Effect of Tanshinone I on the number of TUNEL positive cells in PC-3 and DU145 cells. PC-3 and DU145 cells were treated with Tanshinone I (20, 40 μM) and/or TRAIL (25 ng), and analyzed by TUNEL assay. The fluorescent signals from fragmented DNA (*green*), and DAPI (*blue*) were visualized and photographed by a FLUOVIEW FV10i confocal microscopy. *Magnification* bar = *50 um*. (B) Bar graphs represent quantification of TUNEL positive cells. Data are presented as means ± SEM of triplicate samples. (C) Effect of Tanshinone I and/or TRAIL on apoptotic proteins in PC-3 and DU145 cells. PC-3 and DU145 cells were treated in the absence or presence of Tanshinone I (20, 40 μM) and/ or TRAIL (25 ng/ml) for 24 h. Western blotting was subjected for PARP, procaspase 8, procaspase 9, cleaved caspase3, Bcl-2, Bcl-xL, and β-Actin was used as the internal control. (D) Effect of Tanshinone I and/or TRAIL on p-ERK and p-AKT signaling in PC-3. Western blotting was subjected for p-ERK, total ERK, p-AKT, AKT and β-Actin. Values represent the relative expression of each genes normalized to β- actin by using Image J software. (E) Effect of Tanshinone I (40 μM) and/or TRAIL (25ng) on ROS production by using microplate fluorometer. Hydrogen peroxide (H2O2) was used as a positive control. Data are presented as means ± SEM of triplicate samples. * p < 0.05.

To determine whether the cytotoxicity by co-treatment of Tanshinone I and TRAIL was due to apoptosis induction, FACS analysis and TUNEL assay were carried out in PC-3 or DU145 cells. As shown in Fig 1C, the co-treatment of Tanshinone I and TRAIL increased the population of sub-G1 DNA contents compared to Tanshinone I or TRAIL alone in PC-3 cells. Similarly, the co-treatment of Tanshinone I or TRAIL in DU145 cells increased the population of sub-G1 DNA contents in PC-3 and DU145 cells (Fig 1C) by FACS analysis which was similarly obtained by TUNEL assay (Fig 2A or B). The numbers of TUNEL positive cells were significantly increased by combination of Tanshinone I and TRAIL in both PC-3 and DU145 cells compared to Tanshinone I or TRAIL alone (Fig 2 A and B).

### Combination of Tanshinone I and TRAIL activated PARP and caspase 8, regulated the Bcl-2 family proteins and increased the level of ROS in PC-3 and DU145 cells

Apoptosis is induced through cell death extrinsic pathway or through mitochondrial dependent intrinsic pathway [23]. Western blotting assay showed that combination of Tanshinone I (0, 20, 40 μM) and TRAIL (25 ng) activated caspase 8, caspase 9, caspase 3 and cleaved PARP in PC-3 or DU145 cells (Fig 2C). Furthermore, combination of Tanshinone I and TRAIL suppressed the expression of pro-survival genes such as Bcl-X_L_ and Bcl-2 (Fig 2C) and also attenuated phosphorylation of extracellular signal regulated kinases (ERK), but did not affect p-AKT in PC-3 cells (Fig 2D). In addition, ROS level was determined in PC-3 and DU 145 cells by combination of TRAIL and/or Tanshinone I by fluorescent 2',7'-dichlorofluorescein (DCF) methods. As shown in Fig 2E, Tanshinone I and TRAIL combination increased the level of ROS in PC-3 and DU 145 cells.

### Combination of Tanshinone I and TRAIL upregulated DR5 in PC-3 cells

**Fig 3:**
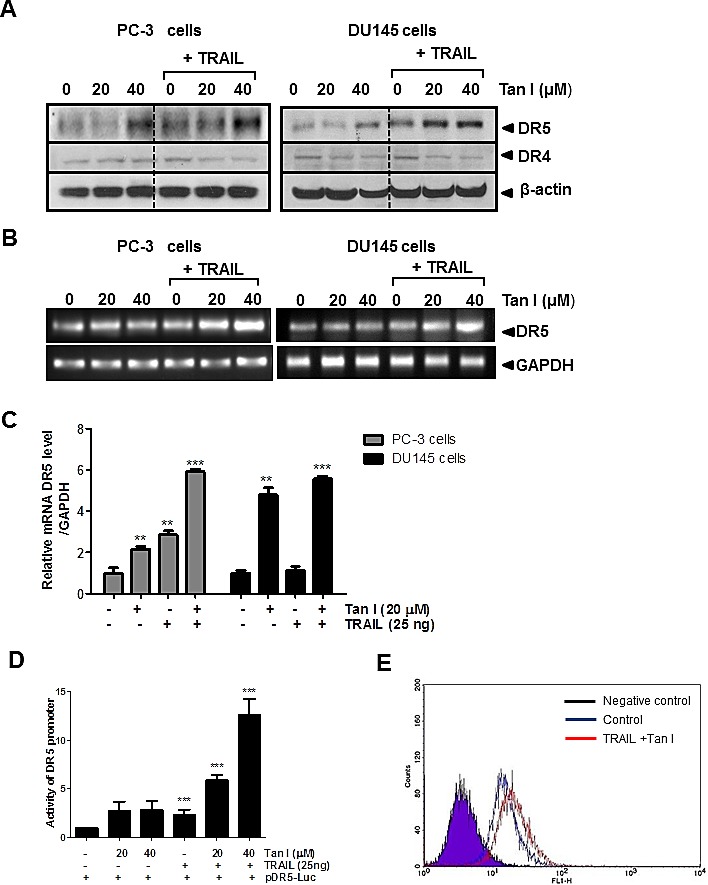
Tanshinone I enhances upregulation of DR5 at mRNA and protein levels, activity of DR5 promoter and cell surface expression of DR5 in PC-3 and DU145 cells (A) Effect of Tanshinone I on DR4 and DR5 at protein level in TRAIL treated PC-3 and DU145 cells. PC-3 and DU145 cells were treated with Tanshinone I (20, 40 μM) and/or TRAIL (25ng/ml) for 24 h. Expression levels of DR5 and DR4 were determined by Western blotting. Beta-actin was used as the internal control. The mRNA expression level of DR5 was determined by RT-PCR (B) or RT-qPCR (C) in TRAIL treated PC-3 and DU145 cells. ** p<0.01, *** p<0.001 vs untreated control. (D) Effect of Tanshinone I on activity of DR5 promoter in TRAIL treated PC-3 cells. DR5 promoter plasmid and *renilla* were transfected into PC-3 cells and incubated for 24 h. Tanshinone I (20, 40 μM) and/or TRAIL (20 ng) were added. Luciferase was normalized to *renilla* luciferase activity. (E) Effect of Tanshinone I on cell surface expression of DR5 in TRAIL treated PC-3 cells by Flow cytometry. Tan I (40 μM) and TRAIL (25 ng) treated PC-3 cells were stained with DR5-FITC conjugated antibody and control IgG (negative control) and analyzed by Flow cytometric analysis.

To understand the apoptotic mechanism of Tanshinone I and TRAIL induced apoptosis, the effect of Tanshinone I and/or TRAIL on the expression of death receptor-related genes such as DR5 or DR4 was evaluated in PC-3 or DU145 cells. As shown in Fig 3A, western blotting showed that combination of Tanshinone I and TRAIL enhanced the expression of DR5 at protein level in PC-3 or DU145 cells, but not DR4. Consistently, combination of Tanshinone I and TRAIL increased the mRNA expression of DR5 by RT-PCR (Fig 3B) or RT-qPCR (Fig 3C) in PC-3 or DU145 cells. To confirm the effect of Tanshinone I and TRAIL on DR5 gene expression, luciferase gene assay was performed. PC-3 cells were transfected with pDR5/-605 promoter and then treated with Tanshinone I and/or TRAIL. Combination of TRAIL and Tanshinone I increased DR5 promoter activity compared to untreated control or TRAIL alone (Fig 3D). Furthermore, FACS analysis for the cell surface expression of DR5 showed that cell surface expression of DR5 was much more detected in Tanshinone I and TRAIL treated PC-3 cells compared to untreated control (Fig 3E).

### Silencing of DR5 suppressed the ability of Tanshinone I and TRAIL combination to enhance cytotoxicity, PARP cleavage and sub G1 population PC-3 cells

**Fig 4:**
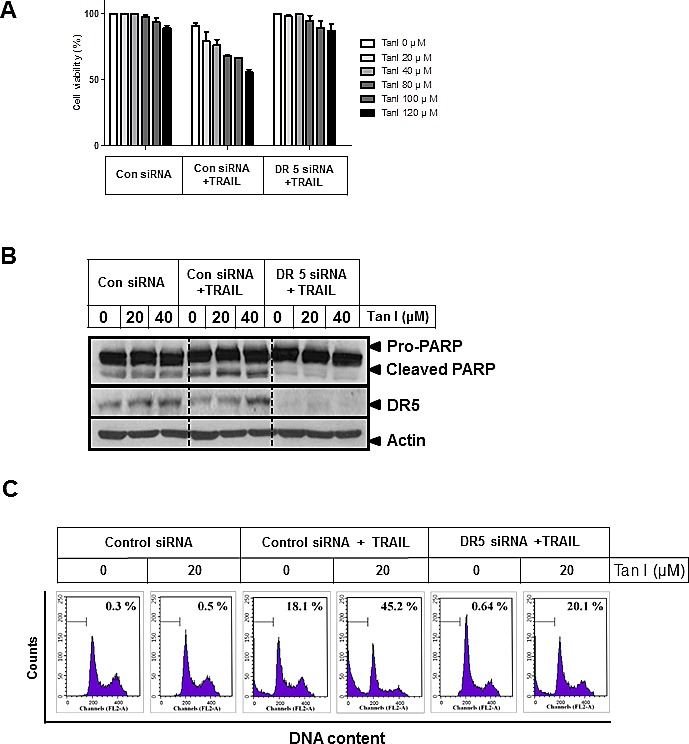
Silencing of DR 5 suppresses cell death induced by Tanshinone I and TRAIL in PC-3 cells PC-3 cells were transfected with control or DR5 siRNA plasmids for 48 h and then treated with various concentration of Tanshinone I (0, 20, 40, 80, 100, 120 μM) and/or TRAIL (25 ng/ml) for 24 h. (A) Effect of DR5 knockdown on the cytotoxicity by combination of Tanshinone I and TRAIL in PC-3 cells by MTT assay. (B) Effect of DR5 knockdown on PARP cleavage and DR5 induced by combination of Tanshinone I and TRAIL in PC-3 cells. Western blot analysis was performed to determine PARP cleavage and DR5 using Actin as an internal control. (C) Effect of DR5 knockdown on increased sub-G1 population by combination of Tanshinone I and TRAIL in PC-3 cells. Flow cytometric analysis was performed for sub-G1 population in DR5 siRNA plasmid transfected PC-3 cells after exposure to combination of Tanshinone I and TRAIL for 24 h.

To determine whether upregulation of DR5 plays a critical role in sensitization of PC-3 cells to TRAIL, control or DR5 siRNA plasmids transfected PC-3 cells were treated with Tanshinone I and/or TRAIL. As shown in Fig 4A, silencing of DR5 suppressed cytotoxicity induced by combination of Tanshinone I and TRAIL, while combination of Tanshinone I and TRAIL exerted dose dependent cytotoxicity in control vector treated PC-3 cells by MTT assay (Fig 4A). Similarly, silencing of DR5 attenuated the cleavage of PARP (Fig 4B) and also reduced sub G1 population up to 20.1% from apoptotic portion (45.2%) exerted (Fig 4C) by combination of Tanshinone I and TRAIL in PC-3 cells.

### Activation of miR135a-3p by combination of Tanshinone I and TRAIL mediated upregulation of DR 5 in PC-3 cells

**Fig 5:**
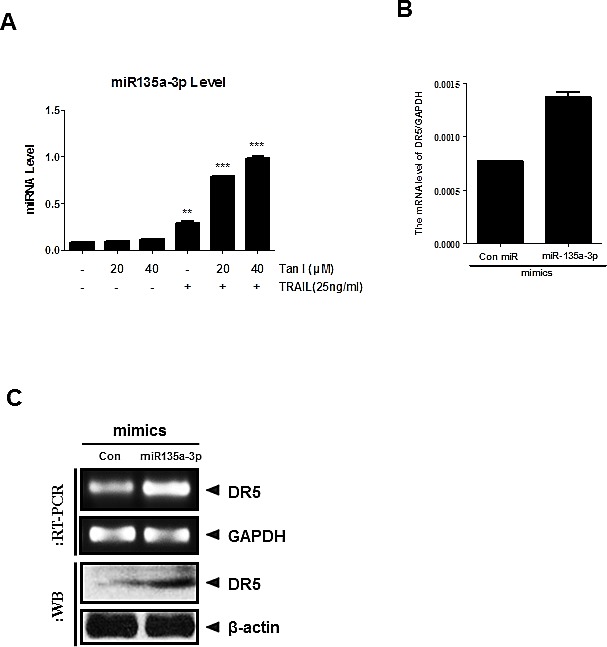
Overexpression of miR135a-3p enhanced sub G1 accumulation and DR5 upregulation at mRNA and protein levels in PC-3 cells (A) Effect of Tanshinone I and TRAIL combination on mRNA level of miR135a-3p in PC-3 cells by RT-qPCR. The expression level of miR135a-3p by combination of Tanshinone I (20, 40 μM) and/ or TRAIL (25ng) was evaluated in PC-3 cells by RT-qPCR. After treatment of Tanshinone I and/or TRAIL in PC-3 cells for 24 h, RT-qPCR was performed with total RNA isolated from PC-3 cells to check the mRNA level of miR135a-3p. (B-C) Effect of miR135a-3p mimics on DR5 at mRNA and protein levels in PC-3 by RT-qPCR (B), RT-PCR (C, upper panel) and Western blotting (C, lower panel).

MicroRNAs, non-coding RNAs, play an important role in apoptosis [24, 25]. MicroRNA 135a was known as a selective killer in glioma cancer cells [20] and a tumor suppressor in renal cell carcinoma [19]. We selected the miR135a for TRAIL combination study among many microRNAs, since bioinformatics analysis through microRNA.org predicted that the binding site of miR135a was at the 3'-UTR of DR5 mRNA. In the current study, we checked whether miR135a-3p mediates apoptosis induced by combination of TRAIL and Tanshinone I in PC-3 cells. Here, the expression of miR135a-3p was upregulated by combination of Tanshinone I and TRAIL in PC-3 cells (Fig 5A). Furthermore, overexpression of miR135a-3p enhanced upregulation of DR5 at mRNA or protein level by RT-qPCR (Fig 5B), RT-PCR and Western blotting (Fig 5C).

### Overexpression of miR135a-3p promoted cytotoxicity, sub G1 accumulation and PARP cleavage in Tanshinone I and TRAIL l cotreated PC-3 cells

**Fig 6:**
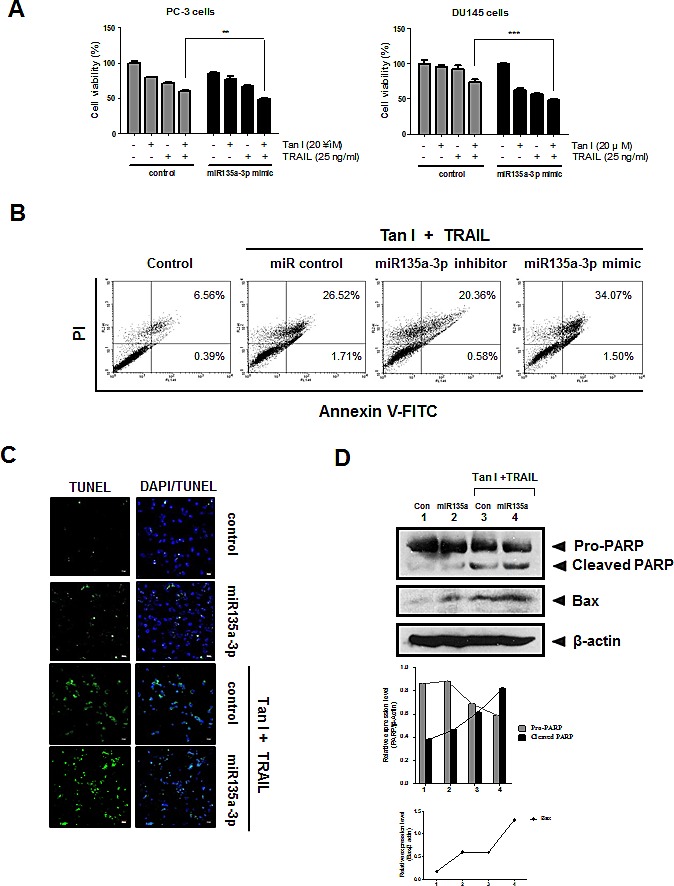
Overexpression of miR135a-3p enhanced cytotoxicity, sub G1 population, the number of TUNEL positive cells, PARP cleavage and Bax in Tanshinone I and TRAIL treated prostate cancer cells (A) Effect of miR135-3p mimics on the cytotoxicity in PC-3 and DU145 cells. Control and miR135-3p mimic plasmids were transfected into PC-3 or DU145 cells for 48 h, and then exposed to Tanshinone I and/or TRAIL for 24 h. Cell viability was determined by MTT assay. ** p<0.01, *** p<0.001 vs combination of Tanshinone I and TRAIL in control. Data are presented as means ± SEM of triplicate samples. (B) Effect of miR135-3p mimics on sub G1 population in PC-3 cells. PC-3 cells transfected with miR135a-3p mimics or inhibitor in the absence or presence of Tanshinone I and TRAIL were stained with Annexin V-FITC and PI (Annexin V-FITC apoptosis detection kit, Sigma). Early and late apoptotic portion was determined by Flow cytometric analysis (FACS analyzer). (C) Effect of miR135-3p mimics on the number of TUNEL positive cells in Tanshinone I and/or TRAIL treated PC-3 cells by TUNEL assay. (D) Effect of miR135-3p mimics on PARP cleavage and Bax in Tanshinone I and/or TRAIL treated PC-3 cells by Western blotting. Bar graphs represent the relative expression of PARP or Bax to β- actin by using Image J software.

To investigate the role of miR135a-3p in cytotoxicity and apoptosis induced by Tanshinone I and/or TRAIL in PC-3 or DU145 cells, miR135a-3p mimic was transfected into PC-3 and DU145 cells and also exposed to Tanshinone I and/or TRAIL. As shown in Fig 6A, miR135a-3p mimic enhanced cytotoxicity by combination with Tanshinone I and TRAIL in PC-3 or DU145 cells compared to Tanshinone I or TRAIL alone. Likewise, we also confirmed that miR135a-3p mimic promoted sub G1 accumulation (34.07 %) induced by combination of Tanshinone I and TRAIL in PC-3 cells compared to Tanshinone I and TRAIL cotreated control (26.52 %), while miR135a-3p inhibitor decreased sub G1 accumulation to 20.36 % (Fig 6B). Also, as shown in Fig 6C, TUNEL assay showed that the numbers of TUNEL positive cells were increased in Tanshinone I and TRAIL cotreated PC-3 cells by miR135a-3p mimic transfection. Consistently, Western blotting revealed that cleaved PARP and BAX by combination of Tanshinone I and TRAIL were enhanced in miR135a-3p mimic transfected PC-3 cells (Fig 6D).

**Fig 7:**
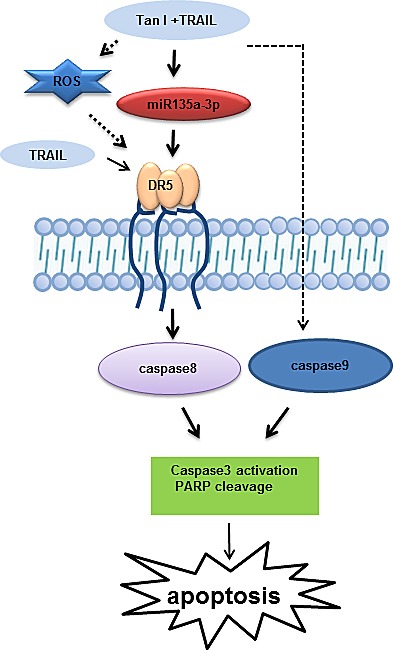
A schematic representation of the signaling pathway affected by combination of Tanshinone I and TRAIL in prostate cancer cells

## DISCUSSION

Though TRAIL has been considered as a potent anticancer agent in several cancers such as colon [6], prostate [26-28], breast [29, 30], bladder and kidney cancers [31, 32], its resistance during cancer therapy is a hot issue to overcome. Thus, recently several natural compounds such as quercetin [33], curcumin [34], artepillin C [35], azadirone [36], emetine [37], crytotanshinone [38] and salirasib [39] are attractive as TRAIL sensitizers in many cancers. In the current study, we provide evidences that Tanshinone I sensitizes the prostate cancer cells to TRAIL induced apoptosis via upregulation of miR 135a-3p mediated DR5.

Combination of Tanshinone I and TRAIL showed synergistic cytotoxicity in PC-3, DU145 and M2182 prostate cancer cells compared to Tanshinone I or TRAIL alone, indicating the potential of Tanshinone I as a TRAIL sensitizer. Also, combination of Tanshinone I and TRAIL cleaved PARP, activated caspase 8/9, and increased sub G1 population and the number of TUNEL positive cells as apoptotic features in PC-3 and DU145 cells, implying that the cytotoxicity of Tanshinone I and TRAIL was induced by apoptosis induction via caspase activation and sub G1 apoptotic portion accumulation.

It is well known that apoptosis is induced via two typical apoptotic pathways such as mitochondrial dependent intrinsic pathway and cell death receptor dependent extrinsic pathway in cancers [40-42]. Also, there are accumulating evidences that overexpression of cell survival or anti-apoptotic proteins such as Bcl-2, and Bcl-xL contributes to TRAIL resistance in cancer cells [43] and TRAIL induces apoptosis by binding to its cell surface death receptors such as DR4 and DR5 [44]. In this regard, our western blotting showed that combination of Tanshinone I and TRAIL attenuated the anti-apoptotic proteins such as Bcl-2, Bcl-_X_L and also activated caspase 8, 9 and DR5 in PC-3 and DU145 cells. We also confirmed the upregulation of DR5 in PC-3 cells by combination of Tanshinone I and TRAIL by promoter assay and FACS analysis for cell surface DR5 expression, demonstrating that Tanshinone I can sensitize prostate cancer cells to TRAIL induced apoptosis via upregulation of DR5 and suppression of survival genes. Conversely, the silencing of DR5 blocked the increased cytotoxicity and sub G1 population and PARP cleavages induced by co-treatment of Tanshinone I and TRAIL in PC-3 cells.

It is well documented that TRAIL induces apoptosis via microRNA regulation [45] including miR34a/ [46] or miR-494 [47] in cancer cells. Thus, Farooqi et al [3] suggested that miRNA regulation of TRAIL-mediated signaling in prostate cancer cells can provide potential biomarkers for the characterization of patients as responders and non-responders for TRAIL-based therapy. Recently, miR-135a was also reported to inhibit the proliferation of renal carcinoma [19], induce apoptosis in gastric cancer cells [48] and promote drug sensitivity in cancer cells[49]. Our RT-PCR and RT-qPCR analyses confirmed that combination of Tanshinone I and TRAIL up-regulated miR135a-3p and DR5 at mRNA level or activity of DR5 promoter in PC-3 cells, indicating the possible involvement of miR135a-3p along with DR5 upregulation in antitumor activity of Tanshinone I and TRAIL in PC-3 cells.

Of note, overexpression of miR135a-3p using its mimic plasmids enhanced the ability of Tanshinone I and TRAIL combination to promote cytotoxicity, upregulate DR5 at mRNA, cleaved PARP and increase the number of TUNEL positive cells in PC-3 cells compared to untreated control, implying that miR135a-3p plays a pivotal role in apoptotic activity by combination of Tanshinone I and TRAIL in PC-3 and DU145 cells.

Collectively, our results demonstrate that combination of Tanshinone I and TRAIL synergistically induce apoptosis via upregulation of miR135a-3p and DR5 in prostate cancer cells and overexpression of miR135a-3p enhances upregulation of DR5 leading to cell death by combined treatment of TRAIL and Tanshinone I. Thus, our findings suggest that Tanshinone I can be used as a potent sensitizer for TRAIL-based combination therapy in prostate cancer cells.

## METHODS

### Cell culture

PC-3, M2182 and DU145 cells (human prostate cancer cells) were purchased from American Type Culture Collection (ATCC, Manassas, USA). The cells were cultured in RPMI1640 medium supplemented with 10% fetal bovine serum (FBS), 2 μM L-glutamine and penicillin/streptomycin of 5% CO_2_ at 37°C.

### Cytotoxicity assay

The cytotoxic effect of Tanshinone I and/or TRAIL against PC-3, M2182, or DU145 cells was evaluated by using 3-(4, 5-dimethylthiazol-2-yl)-2,5-diphenyltetrazolium bromide (MTT) assay (Sigma, St. Louis, MO, USA) according to the manufacturer's instruction. Briefly, prostate cancer cells were seeded onto 96-well microplate and treated with various concentrations of Tanshinone I (0, 20, 40 or 80 μM) (Sigma, St. Louis, MO, USA) and/or TRAIL (25 or 50 ng) (Sigma, St. Louis, MO, USA) for 24 h. MTT solution (5 mg/ml) was added and formazan was dissolved with MTT lysis solution (20% SDS and 50% dimethylformamide). To measure the optical density, microplate reader (TECAN, Austria) at 450 nm was used. Cell viability was determined as a percentage of viable cells in Tanshinone I and/or TRAIL treated group versus untreated control.

### Cell cycle analysis

PC-3 or DU145 cells were treated with Tanshinone I and/or TRAIL for 24 h and fixed in 75% ethanol. Fixed cells were resuspended in PBS containing RNase A (1 mg/ml), and incubated for 1 h at 37°C. After incubation, fixed cells were stained with propidium iodide (50 μg/ml) for 30 min at room temperature in dark for sub G1 population and also for early and late apoptosis, Annexin-V FITC/ propidium iodide double staining was performed according to the manufacturer's instruction. To analyze the DNA contents of the stained cells, CellQuest Software with the FACSCalibur flow cytometry was used (Becton Dickinson, Franklin Lakes, NJ).

### TUNEL Assay

To detect cell death, the DeadEndTM Fluorometric terminal deoxynucleotidyl transferase-mediated dUTP-biotin nick end labeling (TUNEL) system kit was used according to the manufacturer's instructions (Sigma, St. Louis, MO, USA). In brief, DU145 or PC-3 cells treated with Tanshinone I and/or TRAIL for 24 h were washed with cold PBS. Cells were fixed with 4% paraformaldehyde for 30 min and washed twice with PBS for 2 min. Fixed cells in permeabilization solution (0.1%Triton X-100 and 0.1% Sodium citrate) were washed and incubated with TUNEL assay mixture for 60 min. The TUNEL-stained cells were visualized by a FLUOVIEW FV10i confocal microscopy (Olympus, Tokyo, Japan).

### RT-PCR and RT-qPCR analyses

Total RNA from prostate cells was isolated using the QIAzol (Invitrogen, Carlsbad, CA, USA) and one microgram of total RNA was used to make cDNA by Superscript reverse transcriptase and amplified by Platinum *Taq* polymerase with Superscript One Step RT-PCR kit (Invitrogen, Carsbad, CA,USA). Primers sequences used were synthesized by Bioneer (Daejeon, Korea) with the following sequences: hDR5-forwad-5'-GTC TGC TCT GAT CAC CCA AC-3', reverse-5'-CTG CAA ACT GTG ACT CCT ATG-3', hGAPDH -forward - 5'-TCA CCA TCT TCC AGG AGC GA-3'; reverse -5'-CAC AAT GCC GAA GTG GTG GT-3'. For PCR amplification, following conditions was used; an initial step at 50 °C for 30 min, 94 °C for 2 min, followed by 30 cycles at 94°C for 15 s, 55°C for 30 s and 72°C for 1 min, and a final step at 72°C for 10 min. The amplified products were separated on 2% agarose gel. For RT-qPCR, RT-qPCR was performed with the LightCycler TM instrument (Roche Applied Sciences, Indianapolis, IN) with following primers, hDR5- forward: 5'- GAC TCT GAG ACA GTG CTT CGA TGA -3'; reverse- 5'-CCA TGA GGC CCA ACT TCC T-3', hGAPDH-forward5'-CCA CTC CTC CAC CTT TGA CA-3';reverse-5'-ACC CTG TTG CTG TAG CCA -3'.

### Western blot analysis

PC-3 or DU145 cells treated by TRAIL and/or Tanshinone I were lysed in RIPA buffer (50 mM Tris-HCl, pH 7.4, 150 mM NaCl, 1% NP-40, 0.25% sodium deoxycholic acid, 1 M EDTA, 1 mM Na_3_ VO_4_, 1mM NaF and protease inhibitors cocktail). To quantify protein samples, Bio-Rad DC protein assay kit II (Bio-Rad, Hercules, CA, USA) was used. Proteins samples were separated by electrophoresis on SDS-PAGE gel and electrotransferred onto a Hybond ECL transfer membrane (Amersham Pharmacia, Piscataway, NJ, USA). After blocking, the membrane was incubated with primary antibodies for PARP,caspase8, caspase 9, AKT, p-AKT, ERK, pERK, cleaved caspase 3, Bcl-X_L_, Bcl 2, Bax, DR5 and beta actin (Cell signaling, Beverly, MA,USA) followed by exposing to horseradish peroxidase (HRP)-conjugated secondary anti-mouse or rabbit antibodies (AbD serotec, kidlington, UK). To visualize protein bands, chemiluminescence (ECL) system (Amersham Pharmacia, Piscataway, NJ, USA) was used.

### Short interfering RNA (siRNA) transfection assay

PC-3 cells were transiently transfected with control siRNA, or DR5 siRNA (Bioneer, Korea) by using Interferin™ transfection reagent (Polyplus-transfection Inc., New York, NY). Briefly, the mixture of DR5 siRNA (40 nM) and Interferin™ transfection reagent was incubated for 10 min, and added to the cells. The cells were incubated at 37°C for 48 h and treated with Tanshinone I and/or TRAIL or for 24 h.

### DR5 promoter assay

*pDR5/-605* promoter construct (gifted from Dr. TK Kwon, Keimyung University, Korea) was transfected into PC-3 cells along with *Renilla* luciferase reporter plasmid. After 24 h transfection, the cells were treated with Tanshinone I and/or TRAIL for 24 h. Luciferase activity was measured using Dual-Luciferase Reporter Assay System (Promega, Madison, WI, USA).

### FACS analysis for cell surface expression of DR5

To determine the surface expression of the DR5, FACS analysis was carried out. PC-3 cells treated with Tanshinone I and/or TRAIL for 24 h were twice washed with PBS and incubated with 10 μg/ml DR5-FITC conjugated or mouse IgG antibody (Abcam, United Kingdom) in PBS for 1 h at 4°C. After washing, cells were analyzed by flow cytometry using a FACS Calibur flow cytometer (Becton Dickinson, Franklin Lakes, NJ).

### Measurement of reactive oxygen species (ROS) production

PC-3 or DU145 cells were cultured and then pre-incubated with dichloro-dihydro-fluorescein diacetate (DCFH-DA) for 1 h at 37°C. After incubation, DCFH-DA added cells were treated with Tanshinone I and/or TRAIL for 1 h at 37°C. To measure intracellular levels of ROS, the concentration of fluorescent 2',7'-dichlorofluorescein (DCF) was quantified in oxiselect intracellular ROS assay kits (Cell Biolabs, CA, USA) using Fluoroskan Ascent microplate fluorometer (Thomas scientific, NJ, USA).

### MicroRNA (miR) transfection assay

miR135a-3p or control mimic plasmid (200 nM) (Genolution, Korea) was transfected into PC-3 cells using lipofetamine 2000 (Invitrogen, Carlsbad, CA, USA) reagent according to the manufacture's protocol. To evaluate the expression of miR135a-3p, total RNA from PC-3 cells treated by Tanshinone I and/or TRAIL was isolated by QIAzol (Invitrogen). To construct microRNA cDNA, GenoExplorer ™ miRNA cDNA kit (GenoSensor Corporation, Arizona, USA) was used according to the manufacture's protocol. To measure the level of microRNA, RT-qPCR analysis was performed with the LightCycler TM instrument (Roche Applied Sciences, Indianapolis, IN). MicroRNA primers were purchased from GenoExplorer ™ (GenoSensor Corporation, Arizona, USA) and U6 primer was used to normalized the level of microRNAs.

### Combination Index (CI) Calculation

The combination index was performed by Chou-Talalay method and CalcuSyn software (Biosoft, Ferguson, MO, USA). A combination index CI < 1 was considered synergistic [50].

### Statistical analyses

Statistical analyses of the data were conducted using Sigmaplot version 12 software (Systat Software Inc., San Jose, CA). All data were expressed as means ± *standard error* of the mean (*SEM*). The statistically significant differences between control and treatments were calculated by the Student's *t*-test and one-way ANOVA test.
