# Correction: Upregulation of microRNA135a-3p and death receptor 5 plays a critical role in Tanshinone I sensitized prostate cancer cells to TRAIL induced apoptosis

**DOI:** 10.18632/oncotarget.25777

**Published:** 2018-07-17

**Authors:** Eun Ah Shin, Eun Jung Sohn, Gunho Won, Jeong-Un Choi, Myongsuk Jeong, Bonglee Kim, Min-Jeong Kim, Sung-Hoon Kim

**Affiliations:** ^1^ Cancer Preventive Material Development Research Center, College of Oriental Medicine, Kyung Hee University, Seoul, South Korea

**This article has been corrected:** An identical set of Bcl2 bands was mistakenly shown for both the PC-3 and DU145 cells in Figure 2C. The corrected figure for DU145 cells is given below. The authors provided an original Western blot for DU145 cells and declare that these corrections do not change the results or conclusions of this paper.

**Figure 2:**
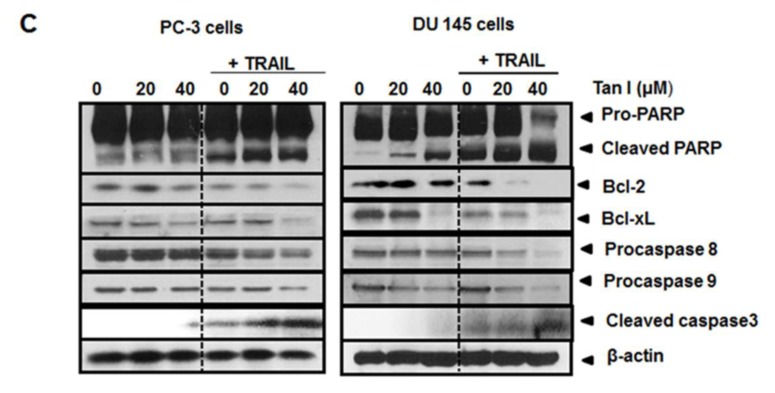
(C) Effect of Tanshinone I and/or TRAIL on apoptotic proteins in PC-3 and DU145 cells PC-3 and DU145 cells were treated in the absence or presence of Tanshinone I (20, 40 μM) and/ or TRAIL (25 ng/ml) for 24 h. Western blotting was subjected for PARP, procaspase 8, procaspase 9, cleaved caspase3, Bcl-2, Bcl-xL, and β-Actin was used as the internal control.

Original article: Oncotarget. 2014; 5:5624-5636. https://doi.org/10.18632/oncotarget.2152

